# An audit of admission clerking of patients in Heddfan, Adult Mental Health Unit in BCUHB - north Wales

**DOI:** 10.1192/bjo.2021.238

**Published:** 2021-06-18

**Authors:** Asha Dhandapani, Sathyan Soundararajan, Manjula Simiyon, Vinila Zachariah, Rajvinder Sambhi

**Affiliations:** BCUHB

## Abstract

**Aims:**

To ensure admission clerking includes salient features needed for the management of both physical and mental health of the patient and also to aid in administrative purposes.

**Method:**

The audit included a team of doctors reviewing the admission clerking notes for 50 patients in the General Adult Psychiatric unit in-patient ward.

We created a standard questionnaire-based on Intended learning outcome of core training in psychiatry CT1-CT3 from Royal College of Psychiatry and standard textbooks.

Our aim is to achieve 100 % compliance in clerking

**Result:**

It was noted that only 30% wrote their GMC number, 4% added route of admission of the patient and a mere 8% filled the Consultants name. Though almost everyone had written the presenting complaints, the other aspects such as history of presenting illness, medical and family history, Allergy status and substance misuse history were missing in many clerking notes. None of them had filled in details of personal history and very few did a risk assessment.

Further lacuna was noted with Mental state examination. Physical examination was also noted to be incomplete. While more than 50% had completed the Blood investigations and ECG, half of them had not documented it and that meant searching in the entire file. A mere 20% filled the nursing observation level whilst none had completed the formulation in the notes.

**Conclusion:**

Admission clerking is a vital source of information that would be needed for the formulation of patients diagnosis and future management.

Apart from this, it also is needed for further continuity of care.

Hence this vital source of information will need to be shared with the junior doctors who will be clerking the patient and seeing them in the first instance.

We, therefore, intend to create a complete clerking proforma along with physical health proforma to aid us in this respect.

We will audit initially in the first round and then plan to introduce a proforma for Clerking and physical examination based on the findings.

We will re-audit to see if the standards are achieved after using the proforma and will consider a Quality improvement project based on this topic

